# Deoxycholic Acid as a Modifier of the Permeation of Gliclazide through the Blood Brain Barrier of a Rat

**DOI:** 10.1155/2013/598603

**Published:** 2013-03-13

**Authors:** Mladena Lalić-Popović, Velibor Vasović, Boris Milijašević, Svetlana Goločorbin-Kon, Hani Al-Salami, Momir Mikov

**Affiliations:** ^1^Department of Pharmacy, Medical Faculty, University of Novi Sad, 2100 Novi Sad, Serbia; ^2^Department of Pharmacology, Toxicology and Clinical Pharmacology, Medical Faculty, University of Novi Sad, 2100 Novi Sad, Serbia; ^3^Faculty of Pharmacy, University of Montenegro Podgorica, 8100 Podgorica, Montenegro; ^4^School of Pharmacy, Curtin University, Perth, WA 6845, Australia

## Abstract

Major problem for diabetic patients represents damage of blood vessels and the oxidative stress of the brain cells due to increased concentration of free radicals and poor nutrition of brain cells. Gliclazide has antioxidative properties and poor blood brain barrier (BBB) penetration. Bile acids are known for their hypoglycemic effect and as promoters of drug penetration across biological membranes. Accordingly, the aim of this study is to investigate whether the bile acid (deoxycholic acid) can change the permeation of gliclazide, through the blood brain barrier of a rat model type-1 diabetes. Twenty-four male Wistar rats were randomly allocated to four groups, of which, two were given alloxan intraperitoneally (100 mg/kg) to induce diabetes. One diabetic group and one healthy group were given a bolus gliclazide intra-arterially (20 mg/kg), while the other two groups apart from gliclazide got deoxycholic acid (4 mg/kg) subcutaneously. Blood samples were collected 30, 60, 150, and 240 seconds after dose, brain tissues were immediately excised and blood glucose and gliclazide concentrations were measured. Penetration of gliclazide in groups without deoxycholic acid pretreatment was increased in diabetic animals compared to healthy animals. Also in both, the healthy and diabetic animals, deoxycholic acid increased the permeation of gliclazide through that in BBB.

## 1. Introduction

Blood brain barrier (BBB) is selective barrier that eclipsed the brain and isolates it from the circulating blood. It is composed of the capillary basement membrane (BM), astrocyte end feet ensheathing the vessels, and pericytes embedded within the BM [1]. It represents a major barrier for drug permeation especially those which are highly hydrophilic and have molecular mass bigger than 400 Da. In addition, efflux protein transporters at the luminal membrane of the endothelial cells limit the penetration of hydrophobic molecules. Diabetes mellitus (DM) can lead to disruption in BBB. A small opening in BBB can have significant impact on BBB function and structure [2]. Some changes in BBB occur very fast after animal being in hyperglycemic state. Previous investigations have shown 30% decrease in brain glucose uptake in only two days after induction of DM [3]. Thus it is viable that changes occur also on some other transport systems but those connected with glucose transport. Damages of central nervous system (CNS) in DM occur due to increased concentration of the free radicals and poor nutrition of the brain cells. Thus, in recent years CNS becomes a target in the treatment of DM [4, 5]. 

Gliclazide is a second-generation sulfonylurea, and it is used in type 2 DM to stimulate insulin production [6]. Sulphonylurea compounds bind to sulphonylurea subunit (SUR) of ATP sensitive K^+^ channel (K_ATP_) in pancreas, which leads to their closure and release of insulin. Gliclazide has selective affinity for binding to SUR1 receptors located mostly in pancreas. Gliclazide is antidiabetic drug with antioxidative properties that are independent of any effect on glucose level [7–9]. Also gliclazide has favorable hemobiological properties and other extrapancreatic effects which make gliclazide potentially useful in type 1 DM as well [10–12]. Because of its scavenging effect and low affinity for binding to SUR receptors in brain gliclazide is a good candidate for the investigation as a protector of brain cells in diabetes. Gliclazide is hydrophobic weak acid that shows low transfer into the brain tissue [13, 14]. 

Bile acids are amphiphilic steroids which have been extensively studied as permeability enhancers of various biological membranes. Bile salts induce reversible BBB opening [15]. Part of the effect is hypothesized to be mediated by tight junction (TJ) modulation [16], cell lysis [17, 18] or incorporation of bile salts into the lipid bilayer [17]. Detergent activity and hydrophobicity are properties of bile salts relevant to the ability to enhance permeability. Deoxycholic acid is natural bile acid that has good permeator capabilities, and it is widely investigated in the field of nanotechnology. 

The aim of this study was to investigate the efficacy of sodium salt of deoxycholic acid (DA) as a BBB permeator of lipophilic molecule gliclazide in healthy and diabetic rats by *in vivo* study of gliclazide uptake into the brain tissue after intra-arterial application of gliclazide by retrograde bolus injection into the right *a. axillaris*.

## 2. Material and Methods

### 2.1. Animal Treatment

#### 2.1.1. Animals

Male *Wistar* rats (aged 2-3 months, weight 200–300 g) were housed under 12 h : 12 h light-dark conditions in an experimental animal facility and have food and water *ad libitum*. All experiments conducted on animals were approved by the Animal Ethic Committee of the University of Novi Sad.

#### 2.1.2. Induction of Diabetes

Diabetes was induced in rats by the intraperitoneal injection of alloxane (100 mg/kg body weight once a day), for two days, as previously described [19, 20]. If hyperglycemia in animals persisted for four days after the second dose of alloxan, animals were included in the experiment. Blood glucose levels were determined from the tail vein blood using a glucometer-strip system (Accucheck Active, Roche). Rats were considered diabetic if blood glucose concentrations were >20 mmol/L [21].

#### 2.1.3. Experimental Protocol

All groups received microsuspension (particle size < 0.5 *μ*m) of gliclazide (20 mg/kg) to the right *a. axillaris.* DA solution (4 mg/kg) was given subcutaneously 30 minutes before gliclazide dose. Before preparing blood vessels, the animals were anesthetized with urethane (1 g/kg) intraperitoneally. Animals were divided into 4 groups: (1) healthy animals given gliclazide (control), (2) healthy animals given gliclazide and DA, (3) diabetic animals given gliclazide, and (4) diabetic animals given gliclazide and DA (6 rats in each group). 

### 2.2. Sample Preparation and Analyses

#### 2.2.1. Serum Samples Preparation

Blood samples were taken from the left *v. jugularis* 30, 60, 150, and 240 seconds after intra-arterial injection of gliclazide and they were centrifuged for 5 minutes (10000 rpm/min). Serums were stored at −20°C until analysed. Acetonitrile was added to serum (in a 2 : 1 ratio) vortexed (30 seconds), and centrifuged for 5 minutes (10000 rpm/min), and then the supernatant (10 *μ*L) was injected into the HPLC system.

#### 2.2.2. Brain Samples Preparation

The animals were decapitated 240 seconds after gliclazide injection. The cranial bones were resected and the brain tissue was divided into: the left and the right cerebral hemisphere (LCH and RCH), brain stem (BS), and cerebellum (C). After being weighed brain parts were stored at −20°C until analysed. Brain parts were homogenized in a 4-fold volume of distilled water. Chloroform was added to brain samples in a 4 : 1 ratio and after vortexing for 30 seconds and centrifuging for 10 minutes on 3500 rpm/min, 1.4 mL of the lower layer was transferred into tubes, evaporated at 70°C, and before injection on HPLC system reconstituted with 100 *μ*L of methanol.

#### 2.2.3. HPLC Determination of Gliclazide

Gliclazide concentrations in serum and brain samples were measured by HPLC system using the modified method of Mikov et al. [21]. HPLC system (*Dionex*) consisted of Agilent column (5 *μ*m, 100 mm × 2.1 mm, 120 A°) with guard column (Agilent; 5 *μ*m, 20 mm × 2.1 mm). The mobile phase consisted of acetonitrile 49% and water 51%, pH 2.7 at flow rate 0.4 mL/min. The retention time for gliclazide was 2.8 minutes. UV detection was set at 229 nm. Analyses were done at room temperature (25°C). The limit of detection (LOD) for brain samples was 0.02 *μ*g/mL and limit of quantification (LOQ) was 0.22 *μ*g/mL with recovery of 87.64 ± 3.63 and for serum samples LOD was 0.2 *μ*g/mL and LOQ was 0.5 *μ*g/mL with recovery of 86.13 ± 3.16%.

#### 2.2.4. Statistical Analysis

All data were reported as mean ± standard deviation (SD). Date were analysed by General Linear Model (Multivariate ANOVA) via SPSS 17.0 (Systat Software Inc., San Jose, CA, USA). Differences were considered significant if *P* ≤ 0.05. Pharmacokinetic analyses were done using WinNonLin (version 4.1; SCI software, Pharsight Corp., Gary NC, USA). 

## 3. Results

Gliclazide concentrations (*μ*g/g) in the brain tissue are shown in Table 1.

Table 1 shows that gliclazide concentrations vary in different parts of the brain and between healthy and diabetic animals suggesting different extent of penetration. There is statistically significant difference of gliclazide concentrations in BS between healthy and diabetic animals without pretreatment with DA. Mean gliclazide concentrations are 7-fold higher in diabetic than those in healthy animals. Also in this part of brain pretreatment with DA leads to the elevation of concentrations of gliclazide in healthy (10-fold) and diabetic rats (3-fold) (differences are statistically significant). 

Gliclazide concentrations in C are 9-fold higher in diabetic than those in healthy animals without DA pretreatment and are statistically different. Pretreatment with DA resulted in higher gliclazide concentration in healthy (10-fold) and diabetic (4-fold) rats, but this differences are not statistically significant.

In the groups treated with gliclazide only, the concentrations of gliclazide in LCH and RCH are lower than those in BS and C. It is noticeable that gliclazide concentrations in RCH are, overall, slightly higher among all treatment group, than its concentrations in the LCH part of the brain. When DA was coadministered with gliclazide, the concentrations of gliclazide in RCH were 3-fold higher in healthy and 4-fold higher in diabetic animals; differences are statistically significant. Gliclazide concentrations in LCH were 4-fold higher in diabetic animals pretreated with DA, while in healthy animals coadministration of DA had no effect on gliclazide concentrations comparing to group that received only gliclazide. 

In total brain mass gliclazide concentrations are elevated in diabetic animals (4-fold, statistically significant) (Table 1). In groups pretreated with DA gliclazide concentrations are 5-fold higher in healthy and 3-fold higher in diabetic animals (differences are statistically significant).

Pretreatment with DA leads to increased variation of concentrations in all groups and brain parts.

Penetration into the CNS was calculated trough formula (1):

(1) $$\begin{array}{c} K_{b/p}\;=\frac{C_{b}}{C_{p}}, \end{array}$$

where *K*_*b*/*p*_ is coefficient of the penetration of gliclazide, *C*_*b*_ concentration of gliclazide in brain tissue (*μ*g/g), and *C*_*p*_ total gliclazide concentration in serum 240 seconds (*μ*g/mL).

Total drug penetration is 24-fold higher in BS in diabetic animals than that in healthy animals given gliclazide alone (Table 2). DA increased penetration of gliclazide in BS 15-fold in healthy and 3-fold in diabetic animals; both differences are statistically significant. Effect of diabetes on penetration in C is more pronounced since drug penetration is 34-fold higher in diabetic than that in healthy animals. In C DA facilitates penetration 48-fold in healthy and 3-fold in diabetic animals (differences are statistically significant). The effect of illness and bile salt on gliclazide penetration through BBB is lower in cerebral hemispheres.

Relative brain mass in group of healthy animals pretreated with DA is 1.2-fold lower than that in group of healthy animals treated only with gliclazide and is statistically different (relative brain mass: 0.69 ± 0.05% Group 1 and 0.56 ± 0.03 Group 2). In diabetic animals pretreated with DA relative brain mass is unchanged in comparison to that of diabetic animals treated only with gliclazide (relative brain mass: 0.60 ± 0.09% Group 3 and 0.62 ± 0.11% Group 4). There are no significant differences in relative brain masses between other groups.

Gliclazide serum concentrations are shown in Table 3. There are different changes in gliclazide serum concentrations during observed period in examined groups. Gliclazide serum concentrations 240 seconds are higher in healthy than those in diabetic animals (statistically significant difference). Also, DA pretreated groups have lower gliclazide serum concentration 240 seconds than groups without DA pretreatment. 

**Figure 1:**
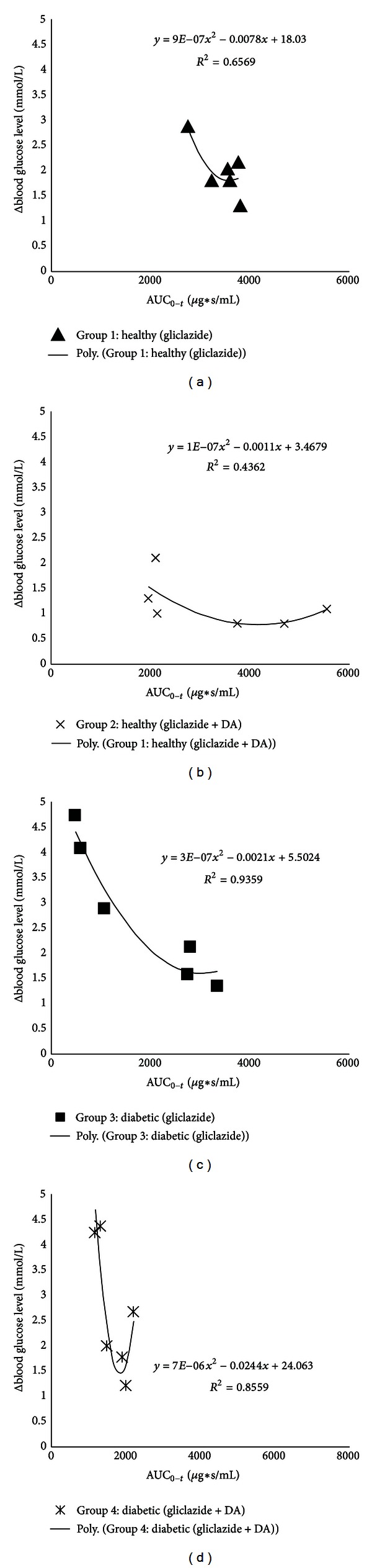
Scatter plots of Δ blood glucose level (mmol/L) 240 seconds after and before intra-arterial injection of gliclazide versus calculated AUC values of gliclazide.

Blood glucose levels (mmol/L) and areas under the curve of gliclazide serum concentrations (AUC) as well as apparent volume of distribution (Vd) are shown in Table 4. Correlation between differences of blood glucose levels 240 seconds after and before application of gliclazide and AUC values is shown on Figure 1. 

Correlation between differences of blood glucose levels and AUCs is shown in Figure 1 and it is second-order polynomial in all groups. When AUC values of gliclazide are higher the differences of blood glucose levels 240 seconds after and before application are smaller. However, after reaching maximum AUC values the differences of blood glucose levels are getting higher again. 

From Table 4 it can seen that DA alone did not change the level of glucose neither in healthy nor in diabetic animals. Treatment with gliclazide in groups of healthy and diabetic animals with and without pretreatment with DA led to an increase of blood glucose levels. Such glucose level changes were not detected in groups of healthy and diabetic animals treated with only saline solution.

Apparent volumes of distribution in the group of healthy animals treated with gliclazide alone are lower than those calculated in other three groups. Healthy animals pretreated with DA had 4.8 fold higher Vd than those in group of healthy animals treated only with gliclazide. Diabetic animals had 1.5 fold lower Vd in group pretreated with bile salt. 

## 4. Discussion

This study showed differences in gliclazide penetration in various parts of the brain under influence of diabetes mellitus and pretreatment with DA. There were higher concentrations of gliclazide in diabetic animals in comparison to healthy animals and they are the result of BBB disruption [22]. Also, DA had a direct permeation-enhancing effect on the antidiabetic drug gliclazide as other bile acids had in previous studies [23–31]. In group of healthy animals pretreated with DA distribution of gliclazide in all brain parts was similar to the one in group of diabetic animals treated only with gliclazide, which indicates that DA as well as diabetes mellitus had direct effect on gliclazide permeability across BBB. 

In healthy animals without DA pretreatment concentrations of gliclazide were similar in all brain parts, which implies a one-compartment model of distribution. In other groups of animals data indicates existence of two compartments where one compartment is cerebellum and brain stem and the other is hemispheres of the brain. However differences in gliclazide brain concentrations could be explained in terms of the distribution of blood vessels and used method of drug application [32]. Higher concentrations of gliclazide in brain stem and cerebellum than those in hemispheres could be due to the fact that gliclazide was given in right *a. axillaris* which directly through *a. vertebralis *takes larger amount of drug to cerebellum and brain stem because they are closer to the place of application, so these regions get the most of the given dose [33, 34]. Also, through the right *a. carotis* drug is taken to the brain hemispheres where it comes first to right cerebral hemisphere. Thus concentrations of gliclazide are higher in right cerebral hemisphere than left cerebral hemisphere. In group of diabetic animals these differences in gliclazide concentrations could be additionally explained with different susceptibility of BBB. It is known that BBB in brain regions with higher blood vessel density and flow, like it is in hemispheres, stays longer intact in hyperglycemic state.

Apparent volume of distribution of gliclazide is higher in diabetic animals than in healthy animals treated only with gliclazide. Also, apparent volume of distribution of gliclazide is higher in those animals pretreated with DA than that in healthy animals treated only with gliclazide. Apparent volume of distribution is in correlation with observed concentrations of gliclazide in brain tissue where volume of distribution was higher in groups of diabetic animals and those pretreated with DA than that in group of healthy animals treated only with gliclazide. Increased distribution in these groups could be explained in various ways. It is known that gliclazide is highly lipophilic small molecule (log⁡⁡*P*_octanol/water_ = 2.6) and weak acid with protein binding of 94% [35]. In diabetes mellitus there is more acidic environment, and thus free fraction of gliclazide is expected to be more in its neutral form which can more easily penetrate BBB [36]. This partly could explain better penetration of gliclazide in group of diabetic animals than in healthy animals. However, the problem of this theory is low solubility of gliclazide which makes it difficult for gliclazide to diffuse through blood to BBB. It is more likely that rapid protein to membrane transfer occurred which was examined earlier for some hydrophobic drugs [37]. If rat brain blood flow is 1.25 mL/min/g and brain blood volume is 3% of brain weight [38] then transit time of blood through brain is approximately 1.5 s. In period of 240 seconds 160 transit times occur thus gliclazide could penetrate through BBB by rapid protein to membrane transfer. 

Diabetic animals have better penetration of gliclazide through BBB than healthy animals. Groups of healthy and diabetic animals pretreated with DA have lower AUCs and higher CNS concentrations. 

There is second-order polynomial correlation of AUCs with differences of blood glucose levels in all groups, where when AUCs are higher the differences of blood glucose levels are smaller till a breaking point when continued increase of AUCs leads to higher blood glucose level differences. This data suggests that too high gliclazide serum levels (AUCs) lead to inefficient blood glucose level control. Also DA changed pattern of the blood glucose level increase and changed polynomial function.

It is shown that DA enhances permeability through BBB in diabetic and healthy animals. Cell lysis is one of the possible mechanism of the BBB opening, which is unlikely to be the case in this investigation since concentration of bile salt at the site was lower than 1.5 mM [17, 36]. Probable mechanism of BBB opening is modification of tight junction or incorporation of bile salt in membrane bilayer. Also, previously it was found that the infusion of bile salts led to opening of the BBB and edemas of the rat brain [39]. In this study brain edemas were not observed since relative brain mass was not higher in groups of animals pretreated with DA. 

## 5. Conclusions

Deoxycholic acid promotes gliclazide penetration across BBB in diabetic and in healthy animals. In addition, deoxycholic acid alters some pharmacokinetic properties of gliclazide in both healthy and diabetic rats. Deoxycholic acid pretreatment also changed the pattern of blood glucose level increase after gliclazide application in diabetic as well as in healthy animals. Thus deoxycholic acid should be more investigated in the treatment of diabetes mellitus and as permeation promoter of lipophilic molecules through BBB as well as other biological membranes.

## Figures and Tables

**Table 1:** Gliclazide concentrations (*μ*g/g) 240 seconds after gliclazide injection (20 mg/kg) in the brain parts and total brain.

Groups		Group 1: healthy Gliclazide *n* = 6	Group 2: healthy Gliclazide + DA *n* = 6	Group 3: diabetic Gliclazide *n* = 6	Group 4: diabetic Gliclazide + DA *n* = 6
Gliclazide concentration (*μ*g/g)—mean ± SD	BS	1.57 ± 0.77	15.49 ± 9.53*	11.51 ± 5.48^#^	33.72 ± 19.58^××,×^
	C	2.22 ± 1.13	23.69 ± 11.45*	19.17 ± 8.18^#^	76.92 ± 34.82^××,##^
	LCH	1.41 ± 0.75	1.87 ± 0.94	0.70 ± 0.38	6.93 ± 4.09^××,×^
	RCH	2.46 ± 1.56	6.33 ± 4.03*	2.88 ± 1.30	10.02 ± 6.43^××,×^
	B	1.71 ± 1.03	9.15 ± 5.53*	7.27 ± 3.41^#^	22.00 ± 14.15^××,×^

LCH: left cerebral hemisphere; RCH: right cerebral hemisphere; BS: brain stem; C: cerebellum, B: total brain.

*Group 2 versus Group 1, *P* ≤ 0.05; ^#^Group 3 versus Group 1, *P* ≤ 0.05; ^×^Group 4 versus Group 3, *P* ≤ 0.05; ^##^Group 4 versus Group 2, *P* ≤ 0.05; ^××^Group 4 versus Group 1, *P* ≤ 0.05.

**Table 2:** Penetration coefficient of gliclazide in the brain tissue after intra-arterial application of gliclazide (*K*_*b*/*p*_).

Groups		Group 1: healthy Gliclazide *n* = 6	Group 2: healthy Gliclazide + DA *n* = 6	Group 3: diabetic Gliclazide *n* = 6	Group 4: diabetic Gliclazide + DA *n* = 6
*K*_*b*/*p*_—mean ± SD	BS	0.09 ± 0.06	1.35 ± 0.92*	2.13 ± 1.41^#^	6.15 ± 4.74^××,×^
	C	0.13 ± 0.06	6.23 ± 3.95*	4.40 ± 2.93^#^	14.05 ± 8.53^××^
	LCH	0.09 ± 0.04	0.11 ± 0.07	0.09 ± 0.04	1.10 ± 0.8^××,×^
	RCH	0.15 ± 0.06	0.46 ± 0.38*	0.32 ± 0.23	1.23 ± 0.98^××,×^
	B	0.12 ± 0.06	1.33 ± 1.01*	1.66 ± 0.93^#^	4.09 ± 2.87^××,×^

LCH: left cerebral hemisphere; RCH: right cerebral hemisphere; BS: brain stem; C: cerebellum, B: total brain.

*Group 2 versus Group 1, *P* ≤ 0.05; ^#^Group 3 versus Group 1, *P* ≤ 0.05; ^×^Group 4 versus Group 3, *P* ≤ 0.05; ^##^Group 4 versus Group 2, *P* ≤ 0.05; ^××^Group 4 versus Group 1, *P* ≤ 0.05.

**Table 3:** Gliclazide serum concentrations (μg/mL) 30, 60, 150, and 240 seconds after intra-arterial injection.

Groups	Time (s)	Group 1: healthy Gliclazide *n* = 6	Group 2: healthy Gliclazide + DA *n* = 6	Group 3: diabetic Gliclazide *n* = 6	Group 4: diabetic Gliclazide + DA *n* = 6
Gliclazide serum concentrations (*μ*g/mL): mean ± SD	30	15.13 ± 4.79	22.77 ± 7.50	12.85 ± 5	9.44 ± 5.72
	60	23.28 ± 4.75	15.37 ± 5.20	10.65 ± 3.61	8.29 ± 4.92
	150	13.94 ± 3.44	10.98 ± 3.75	9.16 ± 4.77	7.22 ± 2.94
	240	15.99 ± 4.26	9.79 ± 3.14*	11.12 ± 4.56^#^	7.43 ± 3.92^××^

*Group 2 versus Group 1, *P* ≤ 0.05; ^××^Group 4 versus Group 1, *P* ≤ 0.05; ^#^Group 3 versus Group 1, *P* ≤ 0.05.

**Table 4:** Areas under the curve of gliclazide serum concentrations (AUC), apparent volume of distribution (Vd), and blood glucose level (mmol/L) 240 seconds after and before intra-arterial gliclazide injection.

Groups	Mean ± SD			
	AUC (*μ*g ∗ s ∗ mL^−1^)	Vd (mL)	Glucose level (mmol/L)	
			Before gliclazide	After gliclazide (240 s)
Group 1 healthy animals: gliclazide *n* = 6	4020.43 ± 456.42	219.73 ± 13.47	7.01 ± 0.3	9.55 ± 0.34**
Group 2 healthy animals: gliclazide + DA *n* = 6	3931.02 ± 1790.13	1077.19 ± 83.43*	8.11 ± 0.60	9.53 ± 0.46**
Group 3 diabetic animals: gliclazide *n* = 6	2150.16 ± 1472.50	970.15 ± 79.97^#^	29.04 ± 4.20	30.95 ± 3.86
Group 4 diabetic animals: gliclazide + DA *n* = 6	1678.7 ± 415.21^××^	648.42 ± 203.65^××^	32.50 ± 5.75	34.3 ± 7.44
Healthy animals	/	/	7.7 ± 0.3	8.7 ± 0.35
Diabetic animals	/	/	28.83 ± 2.23	29.33 ± 2.19
Healthy animals with DA pretreatment	/	/	6.93 ± 0.6	7.45 ± 1.02
Diabetic animals with DA pretreatment	/	/	33.58 ± 4.02	29.7 ± 4.30

*Group 2 versus Group 1, *P* ≤ 0.05; ^××^Group 4 versus Group 1, *P* ≤ 0.05; ^#^Group 3 versus Group 1, *P* ≤ 0.05; **glucose level before versus after gliclazide, *P* ≤ 0.05.

## Abbreviations

AUC: Areas under the curve of gliclazide serum concentrations (*μ*g∗s∗mL^−1^)
BBB: Blood brain barrier
B: Total brain
BS: Brain stem
DM: Diabetes mellitus
*C*_*b*_: Concentration of gliclazide in brain tissue (*μ*g/g)
C: Cerebellum
CNS: Central nervous system
*C*_*p*_: Total gliclazide concentration in serum in 240th second (*μ*g/mL)
DA: Sodium salt of deoxycholic acid
DCH: Right cerebral hemisphere
LCH: Left cerebral hemisphere
TJ: Tight junction
Vd: Apparent volume of distribution (mL).

## References

[1] Ballabh P, Braun A, Nedergaard M (2004). The blood-brain barrier: an overview: structure, regulation, and clinical implications. *Neurobiology of Disease*.

[2] Huber JD, VanGilder RL, Houser KA (2006). Streptozotocin-induced diabetes progressively increases blood-brain barrier permeability in specific brain regions in rats. *American Journal of Physiology*.

[3] McCall AL, Millington WR, Wurtman RJ (1982). Metabolic fuel and amino acid transport into the brain in experimental diabetes mellitus. *Proceedings of the National Academy of Sciences of the United States of America*.

[4] Pocai A, Lam TKT, Obici S (2006). Restoration of hypothalamic lipid sensing normalizes energy and glucose homeostasis in overfed rats. *Journal of Clinical Investigation*.

[5] Sandoval DA, Obici S, Seeley RJ (2009). Targeting the CNS to treat type 2 diabetes. *Nature Reviews Drug Discovery*.

[6] Rendell M (2004). The role of sulphonylureas in the management of type 2 diabetes mellitus. *Drugs*.

[7] O’Brien RC, Luo M, Balazs N, Mercuri J (2000). *In vitro* and *in vivo* antioxidant properties of gliclazide. *Journal of Diabetes and Its Complications*.

[8] Jennings PE, Belch JJF (2000). Free radical scavenging activity of sulfonylureas: a clinical assessment of the effect of gliclazide. *Metabolism*.

[9] Fava D, Cassone-Faldetta M, Laurenti O, De Luca O, Ghiselli A, De Mattia G (2002). Gliclazide improves anti-oxidant status and nitric oxide-mediated vasodilation in type 2 diabetes. *Diabetic Medicine*.

[10] Drouin P, Standl E, Sechser T (2004). Gliclazide modified release: results of a 2-year study in patients with type 2 diabetes. *Diabetes, Obesity & Metabolism*.

[11] Palmer KJ, Brogden RN (1993). Gliclazide: an update of its pharmacological properties and therapeutic efficacy in non-insulin-dependent diabetes mellitus. *Drugs*.

[12] Ziegler O, Drouin P (1994). Hemobiological properties of gliclazide. *Journal of Diabetes and Its Complications*.

[13] Benakis A, Glasson B, Keen H (1980). Metabolic study of 14 C-labelled gliclazide in normal rats and in rats with streptozotocin-induced diabetes. *Gliclazide and Treatment of Diabetes*.

[14] Miyazaki H, Fujii T, Yoshida K (1983). Disposition and metabolism of [^3^H]gliclazide in rats. *European Journal of Drug Metabolism and Pharmacokinetics*.

[15] Mikov M, Kevrešan S, Kuhajda K, Jakovljević V, Vasović V (2004). 3*α*,7*α*-dihydroxy-12-oxo-5*β*-cholanate as blood-brain barrier permeator. *Polish Journal of Pharmacology*.

[16] Deli MA (2009). Potential use of tight junction modulators to reversibly open membranous barriers and improve drug delivery. *Biochimica et Biophysica Acta*.

[17] Greenwood J, Adu J, Davey AJ, Abbott NJ, Bradbury MWB (1991). The effect of bile salts on the permeability and ultrastructure of the perfused, energy-depleted, rat blood-brain barrier. *Journal of Cerebral Blood Flow and Metabolism*.

[18] Yang L, Zhang H, Fawcett JP, Mikov M, Tucker IG (2011). Effect of bile salts on the transport of morphine-6-glucuronide in rat brain endothelial cells. *Journal of Pharmaceutical Sciences*.

[19] Heikkila RE (1977). The prevention of alloxan induced diabetes in mice by dimethyl sulfoxide. *European Journal of Pharmacology*.

[20] Szkudelski T (2001). The mechanism of alloxan and streptozotocin action in B cells of the rat pancreas. *Physiological Research*.

[21] Mikov M, Al-Salami H, Golocorbin-Kon S, Skrbic R, Raskovic A, Fawcett JP (2008). The influence of 3*α*,7*α*-dihydroxy-12-keto-5*β*-cholanate on gliclazide pharmacokinetics and glucose levels in a rat model of diabetes. *European Journal of Drug Metabolism and Pharmacokinetics*.

[22] Tolia E, Fouyas IP, Kelly PAT, Whittle IR (2005). The blood-brain barrier in diabetes mellitus: a critical review of clinical and experimental findings. *International Congress Series*.

[23] Al-Salami H, Kansara H, King J, Morar B, Jayathilaka B, Fawcett PJ (2007). Bile acids: a bitter sweet remedy for diabetes. *The New Zealand Pharmacy*.

[24] Al-Salami H, Butt G, Tucker I, Skrbic R, Golocorbin-Kon S, Mikov M (2008). Probiotic pre-treatment reduces gliclazide permeation (ex vivo) in healthy rats but increases it in diabetic rats to the level seen in untreated healthy rats. *Archives of Drug Information*.

[25] Al-Salami H, Butt G, Tucker I, Mikov M (2008). Influence of the semisynthetic bile acid (MKC) on the ileal permeation of gliclazide in healthy and diabetic rats. *Pharmacological Reports*.

[26] Al-Salami H, Butt G, Fawcett JP, Tucker IG, Golocorbin-Kon S, Mikov M (2008). Probiotic treatment reduces blood glucose levels and increases systemic absorption of gliclazide in diabetic rats. *European Journal of Drug Metabolism and Pharmacokinetics*.

[27] Al-Salami H, Butt G, Tucker I, Mikov M (2008). Probiotic treatment proceeded by a single dose of bile acid and gliclazide exert the most hypoglycemic effect in type 1 diabetic rats. *Medical Hypotheses and Research*.

[28] Al-Salami H, Butt G, Tucker I, Mikov M (2008). Influence of the semisynthetic bile acid MKC on the ileal permeation of gliclazide *in vitro* in healthy and diabetic rats treated with probiotics. *Methods and Findings in Experimental and Clinical Pharmacology*.

[29] Mikov M, Boni NS, Al-Salami H (2007). Bioavailability and hypoglycemic activity of the semisynthetic bile acid salt, sodium 3alpha,7alpha-dihydroxy-12-oxo-5beta-cholanate, in healthy and diabetic rats. *European Journal of Drug Metabolism and Pharmacokinetics*.

[30] Al-Salami H, Butt G, Tucker I, Fawcett JP, Mikov M (2009). Probiotics decreased the bioavailability of the bile acid analog, monoketocholic acid, when coadministered with gliclazide, in healthy but not diabetic rats. *The European Journal of Drug Metabolism and Pharmacokinetics*.

[31] Al-Salami H, Butt G, Tucker I (2009). Gliclazide reduces MKC intestinal transport in healthy but not diabetic rats. *European Journal of Drug Metabolism and Pharmacokinetics*.

[32] Vasović V (1998). *The influence of bile acids derivatives on drug transfer into central nervous system [Ph.D. thesis]*.

[33] Chaisson RB (1973). *Anatomy to the White Rat*.

[34] Hebei R, Stamberg MW (1976). *Anatomy of the Laboratory Rat*.

[35] Remko M (2009). Theoretical study of molecular structure, pKa, lipophilicity, solubility, absorption, and polar surface area of some hypoglycemic agents. *Journal of Molecular Structure*.

[36] Yang L, Zhang H, Mikov M, Tucker IG (2009). Physicochemical and biological characterization of monoketocholic acid, a novel permeability enhancer. *Molecular Pharmaceutics*.

[37] Juliano RL, Daoud S, Krause HJ, Grant CWM (1987). Membrane-to-membrane transfer of lipophilic drugs used against cancer or infectious disease. *Annals of the New York Academy of Sciences*.

[38] Ebling WF, Wada DR, Stanski DR (1994). From piecewise to full physiologic pharmacokinetic modeling: applied to thiopental disposition in the rat. *Journal of Pharmacokinetics and Biopharmaceutics*.

[39] Watanabe A, Fujiwara M, Tominaga S, Nagashima H (1987). Bile acid and ammonia-induced brain edema in rats. *Hiroshima Journal of Medical Sciences*.

